# Uncovering Effects from the Structure of Metabarcode Sequences for Metagenetic and Microbiome Analysis

**DOI:** 10.3390/mps3010022

**Published:** 2020-03-12

**Authors:** David C. Molik, Michael E. Pfrender, Scott J. Emrich

**Affiliations:** 1 Biological Sciences, University of Notre Dame, Notre Dame, IN 46556, USA; mpfrende@nd.edu; 2 Electrical Engineering and Computer Science, University of Tennessee—Knoxville, Knoxville, TN 37996, USA

**Keywords:** minhash, k-mer, microbiome, mantel, PERMANOVA, metagenetics, compression, OTU, ASV

## Abstract

The advent of next-generation sequencing has allowed for higher-throughput determination of which species live within a specific location. Here we establish that three analysis methods for estimating diversity within samples—namely, Operational Taxonomic Units; the newer Amplicon Sequence Variants; and a method commonly found in sequence analysis, minhash—are affected by various properties of these sequence data. Using simulations we show that the presence of Single Nucleotide Polymorphisms and the depth of coverage from each species affect the correlations between these approaches. Through this analysis, we provide insights which would affect the decisions on the application of each method. Specifically, the presence of sequence read errors and variability in sequence read coverage deferentially affects these processing methods.

## 1. Introduction

Sequencing-based analysis of biological communities, also called metagenomics, is increasingly popular. One common approach is to “barcode” sequences from organisms found in a specific location. We define barcoding as the sequencing of a particular genetic locus with the intent of determining taxonomic identity, much like a barcode scanner determines items at a grocery store. When applied to a whole community, this approach is often referred to as metabarcoding. Because barcoded sequences are directly comparable (i.e., are of the same locus), an upfront percent similarity cutoff can be used with de novo clustering to generate an occurrence table that loosely corresponds to species abundances at that location. Sequences that are clustered, or binned, by similarity, are commonly called Operational Taxonomic Units (OTUs). OTUs minimize the effects of slight differences in sequences that may or may not be of interest, as a slight variation could be capturing actual differences between species. Alternatively, Amplified Sequence Variants (ASVs), sometimes called exact sequence variants, are defined as all “unique” reads within a metagenomics dataset and often do not need to be clustered [1]. Because metagenomic data sets are prone to a variety of artifacts resulting from the PCR and sequencing processes, and naturally occurring variation may affect downstream results, additional analysis of the sensitivity of these methods to particular error types and sequence properties is needed. Within studies that do not cluster ASVs, we show that some types of error may become more prominent when compared to OTU-based studies.

Metabarcoding is currently the most cost-effective approach for environmental and biomedical taxonomic surveillance. In practice, barcodes are generated from gene regions that are sufficiently conserved to be PCR amplified across taxonomic groups, but are also variable enough to differentiate branches of the evolutionary tree [2]. Metabarcoding enables the determination of taxonomic diversity in environmental samples where sequences often represent a large number of organisms. This approach is popular because it is often possible to assign sequence bins/clusters (such as OTUs) to any geographic area [3,4]. For example, a recent study used metabarcoding to infer species transfers and inter-relationships in ocean shipping lanes [5]; another used metabarcoding across the Great Lakes to look for invasive species [6]. Another study showed how it could used to make biodiversity assessments [7]. Broader scale applications of metabarcoding can be used to detect seasonal and temporal patterns important for ecosystem restoration and analysis [8,9,10]. We should note that observed differences can result from both large taxonomic differences and from more slight changes that occur between co-occurring related species in an environment [11,12].

Metabarcode analysis often consists of generating sample-to-sample similarities in a pair-wise distance matrix, which can then be processed using either hierarchical clustering [13] or a PERMANOVA statistical analysis [14,15]. Although ASV and OTU analysis have been compared [1,16], to the best of our knowledge no one has looked at the potential effects that data generation of barcode sequences, or their processing from environment to sequencer, have on downstream analysis. Here, we build on previous work presented at the IEEE International Conference on Bioinformatics and Biomedicine in 2018 [17] and evaluate three distinct methods. We created simulated data that incorporate variables that affect real metabarcode analysis: size of conserved regions, which may help fuse closely related clusters; sample depth/coverage, which help detect rare species; and polymorphisms among species that affect all methods. The properties studied are sequencing platform unspecific. As a concrete example, single base-pair errors can be introduced by sequencers [18,19]. By using simulations, we can consider each potential effect independently, and therefore comprehensively test properties that affect environmental metabarcoding analysis.

While we test five different sequence properties, we also analyze three different bioinformatics analysis methods used in metabarcoding. The computing of the ASV method is done using the DADA2 package [20]. The OTUs are created with the QIIME package [3]. The k-mer/minhash method, referred to as the k-mer method, is computed using mash [21]. Because ASVs are determined after sequence trimming and filtering, the prevailing dogma is that the preprocessing will result in observing only the true sequence diversity [16]. In contrast, OTU analysis groups roughly similar sequences together, thus trading some possible species/sequence diversity for higher quality [1]. Lastly, we compare OTUs and ASVs to an alignment-free based method, and specifically to a sparse, random selection-based approach that estimates similarities between samples (k-mer). Although alignment-free methods are not currently utilized in barcoding, they make for an interesting alternative for future studies due to their speed [21].

We show using simulations that the presence of Single Nucleotide Polymorphisms (SNPs) and the number of sequences obtained from each species have effects on the analysis, and that these properties affect the processing methods differently. We first establish that each method can recover a simple simulated structure between samples. Next, we test whether we can recover similar signals between methods from real-world data. Because we are unable to obtain similar results, we then simulate samples with and without common real-world properties to look at variations between the bioinformatics methods considered here. Lastly, we utilize knowledge gained from our initial results in a hybrid approach to better understand how each method would respond to data with a high number of sequence errors. Our results demonstrate that when there is a high number of errors, methods utilizing OTUs or k-mers outperform ASVs.

## 2. Methods

We set up four analyses: a simplified recovery analysis to reveal a simulated community structure; three runs of real-world data analysis; a large number of simulated data runs; and a hybrid approach where SNP errors are injected into real data. Sequences, both simulated and real, are single ended. We process all of these data, both simulated and real, as follows: First, OTU tables are generated from the simulated samples with QIIME [3] using the “pick_open_reference_otus.py” tool and the minimum number of sequences to create an OTU set to one; next we use the ASV method as implemented in the R package DADA2 [20] using default settings, except real-world and hybrid methods where the expected error parameter was reduced to one and all sequences were given high Phred scores. Faked high Phred scores are used in-order to reduce the complexity of the results of the introduced properties. The R package Vegan [14] is then used to generate pair-wise Jaccard matrices from OTU tables. Finally, we consider a sketch-based method based on the “mash” bioinformatics tool, a minhash implementation designed for fast sequence comparisons. Our “mash” analysis uses the default k-mer size of 21 and the default sketch size of 1000 [21].

Simulated data are created with Barcode_Simulator, an in-house script that enables the creation of random DNA sequences, and subsequent species properties (see: Supplement Script 1). The simulated sequences used in this article are modifications of a 500 bp randomized template sequence. Barcode_Simulator can be used to mutate generated sequences to create phylogenetically related sets, change depth of coverage, sequence length, length of conserved regions, and through the use of Run_Simulation.sh, sequence abundance (see: Supplement Script 2). Run_Simulation.sh is a script which takes advantage of the features of Barcode_Simulator to build datasets, which are effectively sets of samples, or sets of sequencing files (see: Supplement Data 1). The simulation pipeline generates a set of sequences that represents a number of different “species”. Each “species” is made up of a number of slightly different sequences, depending on the desired properties.

In our downstream statistical analysis, we make extensive use of the Mantel test [22] to find correlations between distance matrices among samples and among environments [23]. The Mantel test provides a robust statistical tool for multivariate analysis [24]. Our distances matrices are calculated with Jaccard’s distance, so as to not confound how our tested properties could be affecting our results with abundance [25]. We also employ Permutational Multivariate Analysis of Variance (PERMANOVA), which like the more common ANOVA, is used to test whether any coefficient, or mixed coefficient, is a source of variation [15]. We utilize PERMANOVAs when looking for multivariate effects on the differences between samples within real and simulated data sets.

### 2.1. Simulated Community Generation

**Figure 1:**
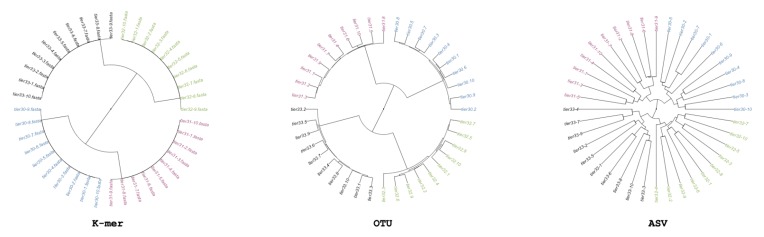
Minhash/k-mer, Operational Taxonomic Units (OTUs), and Amplified Sequence Variants (ASVs)-based dendrograms of simulated “simple” data colored based on source cluster. The groups “tier 3.0” and “tier 3.1” share 10 OTUs, and 3.2 and 3.3 share 10 OTUs. While the imposed structure is recovered by all clusters, the gaps between samples varied with minhash estimating all samples are equidistant, while the most variable is ASV. Mantel correlation between minhash and OTU analysis is 0.97; correlation between minhash and ASV is 0.89; correlation between OTU and ASV is 0.87. While the Minhash/k-mer may look structurally different, its mantel correlation shows that the k-mer method is correlated to the OTU and ASV methods.

We generate simulated community samples as a simple basis for assessing metabarcode-based analysis. We generate these community samples in order to establish that each of the three methods being tested can return the same structure. We generate a single simulated dataset, in which we control the relation of samples so that there is an explicit phylogenetic structure (i.e., some samples are more similar than others). We create 40 samples each containing generalized Next Gen Sequencing (NGS)-like sequences covering 10 unique sequences from a singular pool; next we split the 40 samples into two sets of 20 each receiving an additional 453 sequences from their own pool of 10 sequences; and lastly, both groups of 20 are again split to 10, and each of the four groups receives an additional 453 sequences from their own group of 10 unique sequences. There are 1360 sequences per sample, because samples were generated by randomly picking sequences from the possible sequences at that split; there are only 30 possible different sequences per-group. This simulation scheme produces a quadripartite dendrogram shown in Figure 1.

### 2.2. Real-World Community Data

We also considered previously published 16S-based microbiome datasets derived from baboons from the Amboseli national park in Kenya [26], from soil samples from the Atacama desert [27], and from pitcher plants in the Plumas National Forest in the United States [28]. For these real datasets, the SILVA bacteria dataset was used for the reference sequences for QIIME. For the simulated datasets, the original 500 bp template sequence was added into a closed-reference database. The sequences that did not correspond to a known reference sequence in SILVA were removed prior to further analysis as a standard quality control step [3]. This closed-referenced approach insures the best probability that chosen sequences have the lowest amounts of error. These data were then analyzed with available environmental metadata for the analysis of variance through the use of PERMANOVA.

### 2.3. Simulations of Metabarcode Data

The basic building blocks of our simulations are 500 bp, randomly generated sequences, which are in turn put into sequencing files, which then double as samples. By running entire pipelines multiple times on each dataset, variance resulting from the added properties can be assessed. There are 68 samples in a dataset; each sample is comprised of 136 sequences randomly drawn from a centralized pool of randomly generated sequences. The length, number of sequences, and number of samples are roughly based off of averages of OTU number from metabarcode data from the Atacama desert [27], but are modified later to further explore properties.

Based on an analysis of the Atacama desert microbiome [27], we further explore five specific properties that might affect metagenomic analysis. These properties included the addition of a conserved region (C), variable numbers of SNP polymorphisms (E), variation in the lengths of the sequences (L), variation in the relative abundances of sequences (A), and variation in the total number of sequences per sample (N). The conserved regions are established by the addition of a 24 bp conserved region added at the beginning of each sequence. Variation in SNPs is achieved by the inclusion of up to 10 additional SNP variations in the sequence (the random selection of 1 to 10 polymorphisms is equally likely). The lengths of the sequence recovered are varied from 350 to 500 bp, and variation in the relative abundances of “core” sequences and in the total number of sequences generated is introduced. The baseline model, as an example, selects the following properties: length (L) of all sequences set to 500; 0 SNPs (E); coverage (N) of 1360 sequences chosen from the sequence pool; equally abundant sequences (A); and no conserved sequence (C). The addition of the relative abundance property (A) means that $\frac{1}{6}$ of the base sequences would be a high abundance category, $\frac{2}{6}$ in a middling abundance category, and $\frac{3}{6}$ in a low abundance category (see Table 1 for details). Finally, the number of sequences chosen property (N) implies that the number actual sequences per simulation would vary at random between 140 to 13,600. The analysis pipeline generates a series of 68 random baseline sequences, which are then used to generate simulated samples. All sequences are chosen with equal probability. The combination of, including the absence of, all five properties, produces 32 possible sets for which each has ten simulations, resulting in 320 simulated datasets in total. All possible combinations of properties are analyzed to assess if any compound effects exist.

### 2.4. Simulating Errors in Real-World Data

Utilizing barcoded sequences from the soil samples from the Atacama desert, a random number of SNP errors are added to each sequence in these data. The number of errors is between 1 to 10 in random positions across the sequence. This effect corresponds to the “Added Errors” property of the simulated barcode step, but on real data. Samples were processed in the same way as described for the second step; the error-added Atacama and Atacama soil desert data are correlated with a mantel statistic and PERMONVA applied to see if some analysis of variance can be retained between methods.

## 3. Results

### 3.1. Analysis of Community-Sourced Data

In the initial recovery analysis, in which we simulated inter-related communities, the expected structure is uncovered using all three methods. As shown in Figure 1, OTU, ASV, and minhash-based methods produce nearly identical results. This analysis shows that under ideal conditions, all methods can return similar and expected results.

In the second analysis, when applied to real-world data, these methods are not as well correlated, indicating that there are differences in datasets, which could result from the structures of sequences, and not the sample differences. Using three distinct data sets, we find only a moderate correlation between minhash and more traditional approaches. For example, for the Atacama microbiome dataset we find a mantel correlation of 0.538 between the minhash and OTU methods, 0.231 between the minhash and ASV methods, and 0.409 between the OTU and ASV methods. Running PERMANOVA (see a full list of coefficients used in Table 2, and results in Table 3) indicated that the minhash and OTU methods are significantly (*p*-value < 0.001) affected by overall sample coverage (see: “MBases”, a secondary measure) but the ASV method is not.

**Figure 2:**
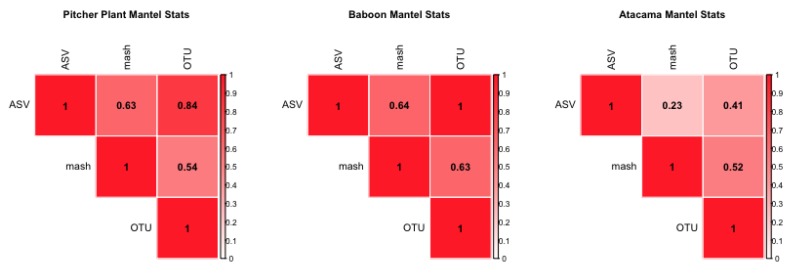
Heatmaps of mantel statistics of environmental datasets; included are the Pitcher Plant data, the Baboon data, and the Atacama data. Generally, the ASV and OTU methods are more correlated to each other than the mash method. Non-one correlations point to differences in the processing methods.

The correlation between OTU and ASV is stronger for the other two datasets considered. We find a mantel correlation for the Baboon data of 0.630 between the OTU and minhash methods, 0.999 between the OTU and ASV methods, and 0.637 between the ASV and minhash methods. (see Figure 2, coefficients tested in Table A1, and results in Table A2) found that the only detectable latent factor is the specific baboon that the microbiome sample was obtained from (i.e., host) and only for the OTU method considered. The Pitcher Plant data analysis is intermediate: the mantel statistic between the OTU and minhash methods is 0.537, between the ASV and OTU methods it is 0.837, and for the ASV and k-mer methods it is 0.628. PERMANOVA analysis (see Figure 2, and results in Supplementary Figure S1–S3).

### 3.2. Uncovering Latent Variables

**Figure 3:**
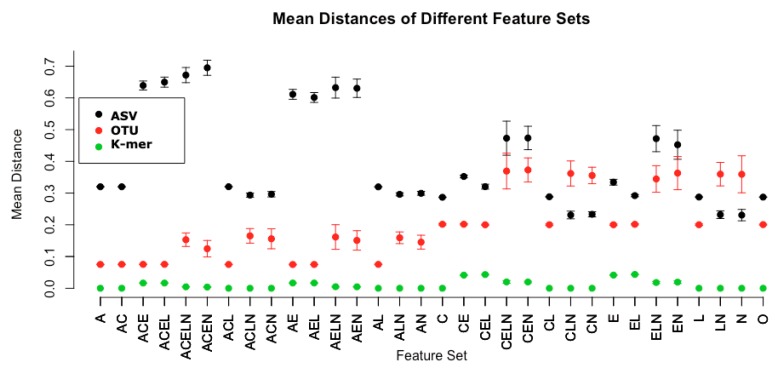
Property set mean distance by method: The average distance across simulated datasets is plotted with error bars by the component properties that make up those data (see Table 1). The presence of E, SNPs, causes distances to go up in ASVs, as expected, and the presence of coverage, N, similarly affects distances and adds variation in OTUs and ASVs. The k-mer method does not seem to have much variation, which suggests that this method is relatively unaffected by either SNPs or coverage.

In the third analysis, to help elucidate and further explore variables that may affect these correlations, but are not uncovered in the real data above, we use simulations. Specifically, we developed simulated sample data that considered all possible combinations of five properties, including the presence of conserved regions (C), SNPs (E), variation in lengths (L), differing abundances (A), and the amount of coverage (N) (see Table 1 in Methods for details.) We find that coverage and SNPs cause notable differences (see Figure 3 for average similarities).

### 3.3. Adding Errors into Real Data

For our final analysis, in which errors were added to real data, the k-mer utilizing method has a correlation between the error induced dataset and the unedited dataset of 0.96; for the OTU method, 0.40; and for the ASV method, 0.06. In the PERMANOVA, while *p*-values changed from processing without the induced errors (see Table A4), they retained signal with the k-mer method, and the OTU method, but signal was lost when using ASVs. We conclude that the addition of errors affects the k-mer and OTU based methods much less than the ASV method.

## 4. Discussion

Simulation at the sequence level is an underutilized exploratory method for determining the properties that affect downstream results in bioinformatics analysis. Environmental metadata could only partially explain the differences observed within real word datasets. More detailed analysis of real and simulated data suggest errors have a large—although slightly different depending on the bioinformatics method used—effect on metabarcode analysis. It is for this reason we can clearly see consistency and expected community structure in our initial simulation, the simplified and induced structure analysis, but not in any other comparison performed in this study.

To begin to determine properties of metabarcode data that do have effects, we performed replicate simulations in which differing coverage could cause differences between methods, especially when some species were rare and required deeper sampling to ensure recovery. This would affect OTU analysis when a Jaccard similarity metric was utilized, because the lack of a rare sequence would affect the similarity computed. Jaccard was chosen for its use in ecology, and that there some indications it would have a reduced effect from sampling error [29]. By creating replicates of datasets with different coverage but the same properties otherwise, we are able to look at how much variation this property (coverage) can cause.

We also found that the sequence coverage property affects the variance of distance matrices across replicates and could manifest in real data when, for example, there was notable primer bias. This is expected: we used Jaccard distance throughout, and this distance metric should affect the ASV method the most, since it does not consider abundances like an alternative such as Bray–Curtis would. To partially overcome this limitation, we hypothesized that explanatory PERMANOVA linear models could help in determining relevant explanatory mixed coefficients, even though there are some simplifications within this analysis; i.e., the generated sequences that are altered to form OTU pools are completely random and we know that this is not the case in real data, as sequences often have some phylogentic relationship to each other. Still, given datasets of sufficient size, the practical differences between Bray-Curtis and Jaccard distances are minimized due to individual abundances becoming less important, and individual effects from our tested properties would be evident [25]. The amount that those properties affected the results would vary given abundance, and the distribution of abundance [29]. Even under simulated conditions, however, we observed a difference of at least 0.06 between computed similarities on the simulated data, on what should be null expectations. While the baseline difference in similarities is not considered significant, it does represent some bound on the precision of the simulation method we use. Significantly, these differences existed in null sets, suggesting that slight differences/induced noise complicate the downstream analysis.

The results of this paper are largely confirmatory; tools that first bin similar sequences, such as OTU methods, and methods that independently look at each sequence, such as ASV methods, are different, especially when additional errors are added. Because the k-mer based method (mash) uses random sampling, it is more resilient to minor differences between sequences. Even though k-mers that underlie minhash sketches could possibly be used to identify species [30,31], the implication of this analysis is that further work is required to use them instead of OTUs. However, a k-mer based or minhash could present a promising tool, and is worth further analysis to determine whether it is a viable bioinformatic tool; this paper starts that analysis. Therefore which of the three methods would actually be chosen for the bioinformatic processing of a metbarcoded dataset is dependent on the analysis desired. Although this paper does not cover any secondary analyses, such as community assembly or community function, the properties studied here would still have effects. Significantly, we show that an increase in read errors within the sequences themselves affects ASV analysis, while the use of OTUs or the k-mer based minhash method is more consistent. While there is some argument into the utility of elucidating properties of OTUs that currently seem to be less preferred than ASVs [1,16], it is important to remember that while ASVs may be increasingly preferred, many times these sequences are grouped into bins of sequences that are representative of different levels of taxonomy, especially for ecosystem function experiments [32]. Binned sequences at different taxonomic levels are analogous to OTUs, and under this regime ASVs would behave like a more traditional OTU-based analysis.

Even so, in light of the differences in behavior of ASVs and OTUs, especially when considering experimental design, the dynamics of ASV and OTU properties should be accounted for in any future experiment. Special attention should be paid to sequence errors in studies that utilize ASVs in non-taxonomic groups.

## Figures and Tables

**Table 1:** Simulated barcode properties.

Property	Abbv.	Description
Differing Abundance	A	Sequences will have differing abundance in each sample i.e.,: High/Med/Low
Conserved Region	C	A conserved region is added to every sequence
Added Errors	E	Errors are randomly added to sequences which make them slightly different
Differing Lengths	L	Sequence lengths are slightly different
Number Chosen	N	Total number of sequences changes per sample
Null Set	O	No properties added

**Table 2:** Coeffcients used in PERMANOVA of the Atacama Microbiome dataset.

Coefficient	Name	Description
MBases_l	Megabases	Number of nucleotide bases in each sample
MBytes_l	Megabytes	Disk space of each sample
AvgSpotLen_l	Sequence Length	Average length of sequences
Air_Relative_s	Air Humidity	Humidity of air on sampling date
Conductivity_s	Soil conductivity	Soil conductivity of sample
Elevation_s	Elevation	Elevation that sample was taken
pH_s	Soil Ph	pH of soil sample

**Table 3:** PERMANOVAs of methods on the Atacama Desert data. Df: degrees of freedom; R2 or Ra^2^: the sum of squares divided by the total; F: Pseudo F, F value by permutation; Pr: P values, based on 9999 permutations.

PERMANOVA of k-mer Method on Atacama					
	Df	SumOfSqs	R2	F	Pr(>F)
MBases_l	1	0.06	0.03	2.69	0.0101
MBytes_l	1	0.04	0.02	1.81	0.0544
AvgSpotLen_l	1	0.09	0.05	4.32	0.0004
Air_Relative_Humidity_s	1	0.44	0.22	20.85	0.0001
Conductivity_s	1	0.06	0.03	2.73	0.0078
Elevation_s	1	0.04	0.02	1.98	0.0367
pH_s	1	0.05	0.02	2.19	0.0244
Residual	59	1.25	0.62		
Total	66	2.02	1.00		
PERMANOVA of OTU method on Atacama					
	Df	SumOfSqs	R2	F	Pr(>F)
MBases_l	1	2.15	0.03	2.31	0.0002
MBytes_l	1	1.48	0.02	1.59	0.0068
AvgSpotLen_l	1	2.84	0.04	3.05	0.0001
Air_Relative_Humidity_s	1	4.36	0.06	4.68	0.0001
Conductivity_s	1	1.34	0.02	1.44	0.0222
Elevation_s	1	1.63	0.02	1.75	0.0031
pH_s	1	1.26	0.02	1.35	0.0380
Residual	59	54.92	0.78		
Total	66	69.97	1.00		
PERMANOVA of ASV method on Atacama					
	Df	SumOfSqs	R2	F	Pr(>F)
MBases_l	1	1.81	0.01	0.82	0.9996
MBytes_l	1	2.24	0.02	1.01	0.4523
AvgSpotLen_l	1	1.78	0.01	0.80	0.9997
Air_Relative_Humidity_s	1	1.66	0.01	0.74	1.0000
Conductivity_s	1	2.04	0.01	0.92	0.9755
Elevation_s	1	2.17	0.01	0.97	0.8239
pH_s	1	2.14	0.01	0.96	0.8854
Residual	59	131.18	0.90		
Total	66	145.01	1.00		

## Supplementary Materials

The following are available at https://www.mdpi.com/2409-9279/3/1/22/s1. Figure S1: *PERMANOVA.PitcherPlant.asv.png*—PERMANOVA table for Pitcher Plant ASV results. Figure S2: *PERMANOVA.PitcherPlant.kmer.png*—PERMANOVA table for Pitcher Plant k-mer results. Figure S3: *PERMANOVA. PitcherPlant.otu.png*—PERMANOVA table for Pitcher Plant OTU results. Script S1: *Barcode_Simulator*— Bash script for generating barcode sequences. Script S2: *Run_Simulation.sh*—Bash script for generating sequence files. Data S1: *simulated_data_matracies.tar.gz*—Tarball of simulated data.

## Appendix A

**Table A1:** Coeffcients Used in PERMANOVA of Baboon Microbiome Dataset.

Coefficient	Name	Description
MBases_l	Megabases	Number of nucleotide bases in each sample
MBytes_l	Megabytes	Disk space of each sample
host_age	Host Age	Baboon’s age at time of sampling
host_sex	Host Sex	Baboon’s sex
host_subject_id	Host UID & Baboon unique identifier	

**Table A2:** PERMANOVAs of methods on Baboon Data.

PERMANOVA of K-mer Method on Baboon					
	Df	SumOfSqs	R2	F	Pr(>F)
MBases	1	0.03	0.00	0.89	0.8486
MBytes	1	0.03	0.01	0.95	0.6047
host_age	1	0.03	0.01	0.97	0.5068
host_sex	1	0.04	0.01	1.18	0.0851
host_subject_id	76	2.81	0.44	1.05	0.0084
Residual	97	3.43	0.54		
Total	177	6.37	1.00		
PERMANOVA of OTU method on Baboon					
	Df	SumOfSqs	R2	F	Pr(>F)
MBases	1	0.64	0.01	0.98	0.4800
MBytes	1	0.59	0.00	0.91	0.6727
host_age	1	0.66	0.01	1.01	0.3945
host_sex	1	0.77	0.01	1.18	0.1324
host_subject_id	76	53.00	0.45	1.07	0.0057
Residual	97	63.39	0.53		
Total	177	119.06	1.00		
PERMANOVA of ASV method on Baboon					
	Df	SumOfSqs	R2	F	Pr(>F)
MBases	1	0.10	0.01	0.96	0.5867
MBytes	1	0.12	0.01	1.16	0.4252
host_age	1	0.09	0.01	0.90	0.6504
host_sex	1	0.06	0.00	0.59	0.8271
host_subject_id	76	6.25	0.38	0.80	0.9982
Residual	97	9.97	0.60		
Total	177	16.60	1.00		

**Table A3:** Coeffcients Used in PERMANOVA of Pitcher Plant Microbiome Dataset.

Coefficient	Name	Description
bp.count	number of base pairs	Number of base pairs in sample
seq..count	number of sequences	Number of sequences in sample
lat*long	latitude and longitude	Latitude and longitude of sample

**Table A4:** PERMANOVAs of methods on Mutated Atacama Desert Data.

PERMANOVA of k-mer Method on Mutated Atacama					
	Df	SumOfSqs	R2	F	Pr(>F)
MBases_l	1	0.06	0.02	1.82	0.0229
MBytes_l	1	0.05	0.02	1.55	0.0479
AvgSpotLen_l	1	0.10	0.04	3.09	0.0005
Air_Relative_Humidity_s	1	0.42	0.15	12.71	0.0001
Conductivity_s	1	0.07	0.02	1.98	0.0141
Elevation_s	1	0.05	0.02	1.50	0.0581
pH_s	1	0.05	0.02	1.54	0.0527
Residual	59	1.97	0.71		
Total	66	2.78	1.00		
PERMANOVA of OTU method on Mutated Atacama					
	Df	SumOfSqs	R2	F	Pr(>F)
MBases_l	1	1.33	0.02	1.23	0.0140
MBytes_l	1	1.33	0.02	1.23	0.0144
AvgSpotLen_l	1	1.80	0.02	1.65	0.0001
Air_Relative_Humidity_s	1	2.95	0.04	2.72	0.0001
Conductivity_s	1	1.12	0.01	1.03	0.3207
Elevation_s	1	1.66	0.02	1.53	0.0002
pH_s	1	1.03	0.01	0.95	0.7059
Residual	59	64.18	0.85		
Total	66	75.42	1.00		
PERMANOVA of ASV method on Mutated Atacama					
	Df	SumOfSqs	R2	F	Pr(>F)
MBases_l	1	2.29	0.01	0.97	0.8383
MBytes_l	1	2.36	0.02	1.00	0.7373
AvgSpotLen_l	1	2.39	0.02	1.01	0.3308
Air_Relative_Humidity_s	1	2.33	0.01	0.99	0.7348
Conductivity_s	1	2.36	0.02	1.00	0.5685
Elevation_s	1	2.34	0.02	0.99	0.7867
pH_s	1	2.22	0.01	0.94	0.9822
Residual	59	139.38	0.90		
Total	66	155.67	1.00		
